# Supplementary material for: RENET2: high-performance full-text gene–disease relation extraction with iterative training data expansion
Source: NAR Genom Bioinform. 2021 Jul 5;3(3):lqab062. doi: 10.1093/nargab/lqab062 (PMC8256824; doi:10.1093/nargab/lqab062)
Supplement: lqab062_Supplemental_File [file lqab062_supplemental_file.pdf]

Supplementary Materials for

RENET2: Accurate Full-text Gene-Disease Relation Extraction with Efficient Iterative Data Expansion

Supplementary Tables

Table S1. Statistics of (a) the abstract datasets (b) the full-text datasets.

(a)

Dataset	Documents	Annotated label			
		Total #	Associated	Ambiguous	Non-associated
Abstract	500	2,813	870	92	1,851
Abstract expansion	500	624	319	47	258
Total	1000	3,437	1,189	139	2,109

(b)

Dataset	Documents	Iteration	Annotated label			
			Total #	Associated	Ambiguous	Non-associated
Full-text	500	1st	793	473	13	307
		2nd	763	421	3	339
		Total	1,556	894	16	646

Table S2. Comparison of RENET2 for relation extraction from abstracts.

Model	Using ambiguous associations	Precision	Recall	F1 score
RENET	No	0.6677	0.7092	0.6878
RENET2	No	0.7117	0.6724	0.6915
	Yes	**0.7127**	**0.7184**	**0.7155**

The best result of each column is in bold.

Table S3. Data and computational resource consumption statistics of RE from 1,889,558 PMC open-access full-text articles.

**Data**	
Number of Full-text	1,889,558

Number of Found GDAs	3,717,569
Raw Data Size	15.4 GB
**Computational resource consumption**	
Total wall-clock	14.65 hours
Total GPU hours	718 hours
GPU Cards (NVIDIA GeForce GTX 1080 Ti)	49

Supplementary notes

The RENET2 network architecture

RENET2 in sequence uses an embedding layer, a convolutional neural network (CNN), a recurrent neural network (RNN), and a fully connected network (FCN) to compute the probability of GDA.

RENET2 uses embedding layers to map token IDs to a vector of features, which consists of two parts: 1) word embedding for each token's context meaning, and 2) role embedding to indicate a token's role in the sentence, including target gene, target disease, non-target gene, and non-target disease. Word embedding and role embedding are concatenated together as the word representation input for the next layer. The input size settings of RENET2 are as follows:

	Abstract	Full-text
Max sentence	32	1000 (basic), 400 (SeFi)
Max tokens per sentence	54	54

RENET2 uses CNN to obtain the sentence feature, BiLSTM to obtain the document feature, and three-layer FCNs, followed by a sigmoid activation, to compute the probability of each GDA. The network architecture layer settings are as follows:

Layers	Size
Token ID embedding	64
Token role embedding	4
CNN kernel size (Conv1D)	[2, 3, 4, 5] X 100
RNN hidden size (BiLSTM)	69 X 2
FCN1	136 X 136
FCN2	136 X 136
FCN3	136 X 1

The hyperparameters used in RENET2 for different types of inputs are as follows:

Hyperparameters	Abstract	Full-text
Learning rate	1e-3	8e-4
L2 penalty term	1e-4	5e-5

Training epochs	18	10
Dropout rate, after token ID embedding	0.3	0.3
Dropout rate, after FCN2	0.1	0.1
